# Extracellular Matrix and Amniotic Derivatives in Bone and Nerve Repair: A Narrative Review of Mechanisms and Their Preclinical and Clinical Applications

**DOI:** 10.7759/cureus.88733

**Published:** 2025-07-25

**Authors:** Zachary Grand, Klaudia Greer, Jonathan Brutti, Christopher Ciesla, Mikaela Rockwell, Jillian Shae, Janae Rasmussen, Payton Frye

**Affiliations:** 1 Medicine, Florida International University Herbert Wertheim College of Medicine, Miami, USA; 2 Orthopedic Surgery, Valley Consortium for Medical Education, Modesto, USA; 3 Medicine, Rocky Vista University College of Osteopathic Medicine, Parker, USA

**Keywords:** amniotic membrane, bone regeneration, decellularized scaffolds, extracellular matrix, mesenchymal stem cells, nerve repair, neurogenesis and neuroplasticity, osteogenesis, perinatal tissue, regenerative medicine

## Abstract

Extracellular matrix (ECM) and amniotic derivatives have emerged as promising biomaterials in regenerative medicine, particularly for bone and nerve repair. These biologic scaffolds provide structural and biochemical cues that support cellular migration, proliferation, and differentiation, thereby facilitating tissue regeneration. ECM-based therapies leverage native bioactive components to modulate immune responses and enhance healing, while perinatal derivatives, including amniotic membrane, umbilical cord, and placental tissue, offer a rich source of growth factors, cytokines, and stem cells that promote neurogenesis and osteogenesis. This literature review explores the mechanistic underpinnings of ECM and perinatal derivatives in bone and nerve repair, detailing their role in cellular signaling, inflammation modulation, and extracellular microenvironment remodeling. Furthermore, we discuss current preclinical and clinical applications, evaluating their efficacy in enhancing functional recovery in orthopedic and neurosurgical contexts. While these therapies hold immense potential, challenges like standardization, immunogenicity, and clinical translation remain key areas for future research. By integrating ECM and perinatal-based approaches, regenerative medicine can advance toward more effective, biologically driven solutions for complex bone and nerve injuries.

## Introduction and background

Bone and peripheral nerve injuries pose substantial clinical challenges, characterized by the inherent difficulty of achieving complete functional recovery. Peripheral nerve injuries affect an estimated 13 to 23 per 100,000 people annually [1], reflecting the significant prevalence of these conditions. Large bone defects resulting from trauma, tumor resection, or degenerative conditions frequently require grafting procedures to restore structural integrity and promote healing. Autologous bone grafts, traditionally harvested from the iliac crest, remain the gold standard for bone regeneration due to their provision of viable osteogenic cells and potent osteoinductive factors [2]. Nevertheless, autograft availability is inherently limited and can result in physical costs to the patient, such as postoperative pain [3,4]. Harvesting procedures carry considerable donor-site morbidity risks, including chronic pain, infection, hematoma formation, and potential fractures [3,4]. While allogeneic bone grafts and synthetic substitutes serve as alternatives, these options often demonstrate inferior integration characteristics or lack the essential bioactive cues necessary for optimal regeneration. This clinical reality highlights the urgent need for biomaterials that can effectively bridge bone defects while minimizing associated complications.

Peripheral nerve injuries, though occurring at a relatively low incidence of less than 1% in the general surgical population, can reach higher rates in specific surgical procedures and patient demographics [5]. When left unrepaired, nerve transections may result in permanent sensory or motor function loss. Surgical nerve repair becomes particularly challenging when gaps between nerve ends preclude direct suture repair. Current practice relies on autologous nerve grafting, typically utilizing sensory nerves, such as the sural nerve, to bridge these gaps; however, this approach necessitates sacrificing healthy neural tissue, creating donor-site deficits, such as numbness, and is constrained by the limited length and number of harvestable nerves [6].

Functional outcomes following nerve autografting remain suboptimal. Many patients achieve only partial sensory or motor recovery even after technically successful repairs, particularly in cases involving proximal injuries or extensive gaps [7]. Synthetic nerve conduits, constructed from materials such as collagen or polymers, have been used to bridge small gaps (typically ≤3 cm), but their effectiveness diminishes markedly as gap length increases [8]. These limitations highlight the critical need for improved nerve reconstruction strategies that promote superior regeneration without incurring additional patient morbidity.

Extracellular matrix (ECM)-based scaffolds, particularly those derived from human perinatal tissues, such as the amniotic membrane (AM) and umbilical cord, have gained recognition as promising adjunctive therapeutic options for bone and nerve repair. These biologically derived materials provide a natural three-dimensional architecture enriched with bioactive molecules that facilitate cell attachment, migration, and subsequent tissue regeneration. Unlike permanent synthetic implants, ECM scaffolds typically exhibit biodegradable properties that enable integration with host tissues while avoiding long-term foreign body responses. Perinatal-derived biomaterials are particularly appealing due to their abundant growth factor content and inherent immune-privileged characteristics. The human AM exemplifies this potential, containing more than 30 identified cytokines and growth factors that actively modulate inflammatory responses and promote healing processes [9].

In this review, we aim to synthesize recent advances in ECM- and amniotic-based therapies for bone and nerve regeneration, underscore how these biologic approaches compare with traditional grafting techniques, and identify the key challenges and future directions required to translate these strategies into widespread clinical practice. The following sections examine the multifaceted role of ECM in bone and nerve repair, exploring its composition, regenerative mechanisms of action, and current clinical applications of ECM-based scaffolds in these therapeutic contexts.

## Review

Methodology

We conducted a comprehensive literature search to gather relevant publications on ECM and amniotic-derived therapies for bone and peripheral nerve repair. The search included PubMed-indexed journals and databases (e.g., MEDLINE) covering the period 1965-2025, using keywords such as "extracellular matrix scaffolds," "amniotic membrane," "bone regeneration," and "nerve regeneration." Both preclinical (in vitro and animal) studies and clinical studies (including case series, cohort studies, and clinical trials) were considered. There were no strict language exclusions, although the vast majority of sources were in English. Articles were selected for inclusion based on relevance to the topic and their contribution of significant data or insights into mechanisms or clinical outcomes. Because this is a narrative (nonsystematic) review, we did not perform a formal risk-of-bias assessment or meta-analysis of pooled data. Instead, we qualitatively synthesized the evidence, highlighting higher levels of evidence (e.g., randomized trials or systematic reviews) where available, and noting the descriptive nature of lower-level evidence (case reports, preliminary studies) when interpreting results. This approach provides a broad overview of the field while acknowledging that conclusions are limited by the heterogeneity and primarily early-stage nature of available data.

The ECM in bone and nerve repair

Structure and Composition of ECM

The ECM serves as the fundamental structural framework of tissues, comprising a sophisticated network of proteins, glycosaminoglycans, and tissue-specific macromolecules [10]. Bone ECM exhibits remarkable specialization, with a majority consisting of inorganic hydroxyapatite minerals [11]. In comparison, the organic component is predominantly type I collagen (~90%), supplemented by minor collagens such as type V, non-collagenous proteins including osteocalcin and osteopontin, and sequestered growth factors [12]. This mineralized collagenous architecture grants mechanical strength to bone while simultaneously functioning as a reservoir for critical signaling molecules. The seminal work by Urist demonstrated this principle of demineralized bone matrix-induced ectopic bone formation, revealing the presence of bone morphogenetic proteins (BMPs) embedded within the ECM [13]. Therefore, bone ECM transcends its role as a mere scaffold for osteoblasts and osteoclasts, providing essential instructive signals for osteogenesis.

Perinatal tissue-derived ECM maintains structural similarities to adult matrices while exhibiting distinctive enrichment in regenerative factors. The human AM exemplifies this duality, featuring a basement membrane containing collagen IV, laminin, and fibronectin on one surface, and a stromal matrix rich in collagens I and III, elastin fibers, and glycosaminoglycans on the opposite side. The absence of vascular structures and immune cells contributes significantly to its low immunogenic profile. Comprehensive biochemical analyses of processed amniotic and chorionic membranes have revealed an extensive repertoire of growth factors, including basic fibroblast growth factor, vascular endothelial growth factor (VEGF), and transforming growth factor-β (TGF-β), alongside various cytokines embedded within the ECM [9]. Similarly, Wharton's jelly from umbilical cord tissue represents an ECM-abundant gelatinous structure with elevated concentrations of hyaluronan, collagens, and sulfated proteoglycans, creating a supportive microenvironment for umbilical vessels [14,15]. These unique compositional characteristics of perinatal ECM suggest considerable therapeutic potential, offering both scaffolding properties and bioactive signals that facilitate tissue regeneration when utilized as graft materials.

Mechanisms of Action in Regeneration

ECM-based scaffolds facilitate tissue repair through three primary mechanisms: structural support, biochemical signaling, and immune modulation [16,17]. In bone repair applications, natural ECM scaffolds, particularly collagen-based matrices, can fill critical-sized defects and support the migration and attachment of osteoprogenitor cells [18]. Their inherent porosity promotes vascular infiltration and enables new bone deposition throughout the scaffold architecture [19]. For nerve repair, the preserved basal lamina structure of decellularized nerve grafts provides essential physical guidance cues that direct regenerating axons across defects, mimicking the function of autologous nerve grafts by creating organized pathways for axonal extension [20]. The aligned collagen and laminin organization within these grafts maintains axonal directionality and facilitates proper reconnection to distal targets.

Beyond passive structural support, ECM scaffolds exhibit significant bioactivity through biochemical signaling pathways that influence cellular behavior [21]. In bone tissue, endogenous growth factors, such as bone morphogenetic protein-2 (BMP-2) and TGF-β, embedded within the matrix, become gradually available to stimulate mesenchymal stem cell recruitment and subsequent differentiation into functional osteoblasts [13]. This osteoinductive capacity represents a key advantage of natural bone-derived matrices over synthetic alternatives. In peripheral nerve applications, ECM components, such as laminin and fibronectin, engage integrin receptors on Schwann cells and other neurons, activating intracellular signaling cascades that promote axonal sprouting and elongation [22].

The immunomodulatory properties of ECM scaffolds represent an equally critical mechanism of action. Unlike permanent synthetic implants, which often elicit chronic inflammatory responses or fibrotic encapsulation, properly processed decellularized ECM materials typically promote constructive tissue remodeling. Preclinical studies have shown that implanted biological scaffolds can favorably shift macrophage phenotypes from pro-inflammatory M1 profiles toward pro-regenerative M2 phenotypes [23]. This M2-dominant response correlates with increased anti-inflammatory cytokine release, enhanced matrix remodeling, and the development of functional tissue rather than fibrotic scarring. Perinatal tissues possess inherent anti-inflammatory properties. For example, AMs contain interleukin-10 and various fibrosis suppressors that actively reduce scar formation [24,25]. This feature helps explain the clinical success of AM grafts in preventing adhesions following tendon and nerve surgeries.

In summary, ECM scaffolds support tissue regeneration through multifaceted mechanisms that extend beyond bridging physical defects. These biological materials actively contribute to healing by delivering essential cues for cellular differentiation and growth, while also shaping immune environments that promote effective tissue integration and functional recovery.

To clarify the differences between ECM derived from adult versus perinatal sources, we summarize key structural and functional attributes in Table 1.

**Table 1:** Comparison of Adult-Derived Versus Perinatal-Derived ECM ECM: extracellular matrix; IL-10: interleukin 10; TGF-β: transforming growth factor-beta; VEGF: vascular endothelial growth factor; BMP: bone morphogenetic proteins

Characteristic	Adult-Derived ECM	Perinatal-Derived ECM
Source	Bone, dermis, tendon, nerve (adult tissues) [11]	Amniotic membrane, umbilical cord, Wharton's jelly, placenta [9,14,15]
Structural Composition	Predominantly collagen I, variable matrix proteins [11]	Rich in collagen III, IV, hyaluronic acid, proteoglycans [14,15]
Growth Factor Content	Moderate, context-dependent [26]	High; includes IL-10, TGF-β, BMPs, VEGF [9,14,22,24]
Regenerative Potential	Supports healing, limited stem cell content [26]	High regenerative and immunomodulatory capacity [15,27,28]
Immunogenicity	Moderate (depends on decellularization) [24,27]	Low (immune-privileged tissue) [14,27]
Mechanical Properties	Dense, biomechanically mature [11]	Flexible, gel-like, hydrated [14-16]
Common Clinical Applications	Orthopedic grafts, nerve conduits [11]	Wound healing, nerve wraps, bone regeneration scaffolds [29,26]

Extracellular and amniotic derivatives in bone and nerve repair

Perinatal tissues, such as the human AM, umbilical cord, and placenta, have emerged as rich reservoirs of ECM and cellular components that confer significant regenerative potential [27,28]. These biologic derivatives provide native collagenous scaffolding along with a cargo of growth factors, cytokines, and mesenchymal stem cells, creating a pro-healing microenvironment conducive to both osteogenesis and neuro-regeneration [29,26]. Unlike synthetic materials, amniotic and placental grafts inherently modulate the wound's environment by releasing bioactive signals that recruit host cells and promote their differentiation into bone-forming osteoblasts or regenerating Schwann cells and neurons [30]. The low immunogenicity of these perinatal matrices, due to minimal human leukocyte antigen (HLA) class II expression, permits allograft use with a reduced risk of immune rejection, underscoring why these derivatives are being explored as off-the-shelf solutions for complex bone defects and nerve injuries [31]. By leveraging the innate biological richness of birth tissues, researchers aim to overcome the limitations of conventional grafts using materials that actively orchestrate tissue repair rather than merely filling voids within the injured tissue. This paradigm shift positions perinatal-derived biomaterials as a bridge between cell therapy and traditional scaffolds, potentially enabling regenerative therapies that integrate seamlessly into the host environment.

A key mechanism by which amniotic and other perinatal derivatives enhance healing is through potent immunomodulation and fibrosis attenuation at injury sites. The AM secretes factors that suppress pro-inflammatory cytokines and downregulate TGF-β-driven fibrotic pathways, thereby reducing scar tissue formation in regenerating nerve and bone tissues [28,26]. In peripheral nerve repair, wrapping injured nerves with human AM has been shown to significantly reduce perineural adhesions and inhibitory scar formation, creating a permissive channel for axonal regrowth [26,30]. Simultaneously, these derivatives release a broad spectrum of growth factors, including angiogenic and neurotrophic factors, that stimulate cell proliferation and guide the regeneration of vascular and neural networks within healing tissues [28,30]. Amniotic mesenchymal and epithelial cells also contribute paracrine signals (e.g., epidermal growth factor (EGF), VEGF, and nerve growth factor (NGF)) and extracellular vesicles that further orchestrate repair processes from promoting osteoblastic activity in bone defects to enhancing Schwann cell migration and axon extension across gaps in a nerve [29,30]. Notably, the intrinsic ECM of these grafts provides a favorable substrate for cellular attachment and matrix remodeling, integrating with host tissue while gradually resorbing in vivo. This dynamic interplay of immune modulation, trophic support, and scaffold function underlies the multifaceted regenerative actions of perinatal-derived biomaterials. By mitigating the hostile post-injury milieu and actively directing tissue repair at the molecular level, these biologics help recapitulate a healing response that more closely resembles fetal regenerative capacity than the healing potential achieved with conventional implants.

Extensive preclinical evidence supports the efficacy of amniotic and placental derivatives in enhancing bone and nerve repair, validating their proposed mechanisms of action. In models of large bone defects, the application of AM allografts, either as membrane wraps or cell-seeded scaffolds, consistently accelerates new bone formation and mineralization compared to injuries treated with standard collagen membranes or left unfilled [29]. Amniotic-derived cells can differentiate along the osteogenic lineage and secrete osteo-inductive signals, resulting in more robust and organized bone regeneration in critical-sized defects, with outcomes approaching those achieved using the Masquelet (induced membrane) technique employed clinically for massive bone loss. The induced membrane technique, which involves staged reconstruction using a biologically active membrane formed around a cement spacer, has been extensively studied and optimized with clinical success in long bone reconstruction, demonstrating reliable union rates even in challenging anatomical and infectious contexts [32]. Similarly, in peripheral nerve injury models, conduit or wrap devices incorporating human AMs have led to increased axon counts, faster recovery of nerve conduction, and improved motor function relative to controls [30]. Notably, a systematic review of preclinical studies found that nerves repaired with amniotic wraps showed significantly better functional outcomes in most reports, highlighting the reproducible benefits of the amnion's anti-fibrotic and neurotrophic effects [26]. Early clinical applications are beginning to reflect these findings. For example, AM allografts have been successfully used in oral and orthopedic surgeries to augment bone healing, and in nerve decompression and repair procedures to prevent scar-related complications without significant immune-related adverse events [33]. These outcomes highlight the direct regenerative contributions of perinatal tissue matrices and their role in supporting the body's intrinsic repair mechanisms. Table 2 summarizes the key biologics used in bone and nerve repair, detailing their specific mechanisms of action in both therapeutic contexts.

**Table 2:** Key Biologics for Bone and Nerve Repair: Examples and Mechanisms of Action ECM: extracellular matrix; 3D: 3-dimensional; TGF-β: transforming growth factor-beta; EGF: epidermal growth factor; VEGF: vascular endothelial growth factor; BMP: bone morphogenetic proteins; rhBMP-2: recombinant human bone morphogenetic protein-2; Trk: tyrosine kinase; GDNF: glial cell line-derived neurotrophic factor; GFRα: GDNF family receptor-alpha; NGF: nerve growth factor; BDNF: brain-derived neurotrophic factor; MSC: mesenchymal stem cell; PRP: platelet-rich plasma; PDGF: platelet-derived growth factor; IGF-1: insulin-like growth factor-1

Biologic Type	Mechanisms in Bone Repair	Mechanisms in Nerve Repair
Decellularized ECM Scaffolds	Acellular tissue matrices (e.g., decalcified bone or decellularized nerve grafts) provide a natural 3D collagenous scaffold that is osteoconductive, guiding new bone growth into the defect [34]. Importantly, these matrices retain native growth factors (such as bone morphogenetic proteins) and bioactive cues, imparting osteoinductive properties that stimulate progenitor cell differentiation and bone formation [34]. With cellular antigens removed, they elicit minimal immune response while promoting "constructive" remodeling of bone tissue.	Removing cellular antigens from nerve or other tissues yields a scaffold with preserved extracellular architecture (e.g., endoneurial tubes or basement membrane) that guides regenerating axons [35]. The decellularized ECM retains essential laminin, collagen, and glycosaminoglycan components that support Schwann cell migration and adhesion of regenerating neurons [35]. These grafts lack immunogenic cells, but are rich in native ECM signals and pro-angiogenic factors. These grafts provide a permissive 3D substrate for axon extension across nerve gaps and encourage revascularization of the repair site.
Perinatal Tissue Derivatives (e.g., Amniotic Membrane)	Amniotic membrane and related perinatal tissues (placenta, Wharton's jelly) serve as biologically active scaffolds that release a rich cocktail of growth factors and cytokines to stimulate osteogenesis [36]. They are inherently anti-inflammatory and anti-fibrotic, dampening local inflammation and scar formation while their resident mesenchymal stem cells and bioactive matrix promote new bone deposition [37]. Used as graft wraps or membranes, perinatal tissues create a favorable milieu for bone regeneration, enhancing healing of fractures and defects by recruiting osteoprogenitor cells and modulating the immune response.	Perinatal-derived biomaterials provide a neuroprotective, pro-regenerative environment for injured nerves. The human amniotic membrane, for example, exhibits low immunogenicity and secretes numerous growth factors (e.g., TGF-β, EGF, VEGF) and neurotrophic factors that support neural outgrowth [30]. When applied as a nerve wrap or conduit, amniotic tissue reduces fibrosis and inflammation around the injury, acting as an anti-adhesive barrier. It also delivers mesenchymal progenitor cells and trophic factors that foster axonal regeneration and remyelination, effectively creating a permissive microenvironment for nerve repair [30].
Growth Factors (Signal Molecules)	Osteogenic growth factors such as BMPs and TGF-β are potent drivers of bone repair. BMP-2, in particular, binds to mesenchymal stem cell receptors to induce osteoblast differentiation and robust bone matrix production [38]. These factors orchestrate the bone healing cascade by stimulating osteoprogenitor proliferation, enhancing angiogenesis, and upregulating osteocalcin and collagen synthesis for mineralized matrix formation. Clinically, the exogenous delivery of BMPs (e.g., rhBMP-2 in grafts) has been shown to significantly augment fracture healing and spinal fusion by recapitulating embryonic bone formation pathways.	Neurotrophic growth factors (e.g., nerve growth factor, brain-derived neurotrophic factor, glial cell line-derived neurotrophic factor) are essential for peripheral nerve regeneration. They bind to specific neuronal receptors (Trk receptors or GFRα co-receptors) to activate intracellular pathways that promote neuron survival and axon elongation [30]. After nerve injury, endogenous levels of these factors rise but are often insufficient; thus, sustained exogenous supply can markedly improve regeneration [39]. Incorporating growth factors into nerve conduits or injection sites enhances axonal outgrowth and guidance, as demonstrated by improved nerve fiber extension and functional recovery when NGF, BDNF, or GDNF are delivered to repairing nerves.
Mesenchymal Stem Cells (MSCs)	MSC-based therapies aid bone repair through both direct and paracrine mechanisms. These multipotent stromal cells can engraft into bone lesions and differentiate into osteoblasts that deposit new bone matrices. However, an equally critical role of MSCs is immunomodulation: they secrete anti-inflammatory cytokines and growth factors that orchestrate the fracture healing process [40]. By modulating macrophage and T-cell responses at the injury site, MSCs help resolve inflammation and stimulate vascularization and tissue remodeling [40]. The net effect is an enhanced regenerative microenvironment that accelerates bone union, even when only a small fraction of the cells directly become new bone-forming osteocytes.	Transplanted MSCs promote peripheral nerve regeneration primarily via supportive paracrine effects and glial-like activity. Even without full differentiation, MSCs secrete a broad spectrum of neurotrophic factors, angiogenic factors, and extracellular vesicles that collectively support axonal growth and neuronal survival [41]. They can adopt Schwann cell–like phenotypes under appropriate cues, wrapping and remyelinating regenerating axons. Owing to their low immunogenic profile, even allogeneic MSCs survive well in nerve repair sites and continuously release factors that guide axons and suppress hostile inflammation [41]. In experimental nerve injuries, MSC therapy has yielded faster and more complete functional recovery by filling nerve conduits with a pro-regenerative cellular substrate.
Platelet-Rich Plasma (PRP)	PRP is an autologous concentrate of platelets that, upon activation, releases a plethora of growth factors pivotal for bone healing (PDGF, TGF-β, IGF-1, VEGF, etc.). These signaling molecules synergistically accelerate the repair cascade: they promote angiogenesis, collagen deposition, extracellular matrix formation, and the recruitment and proliferation of osteoblast progenitors [42]. PRP thus jump-starts the normal fracture healing stages - inflammation, soft callus, mineralization - often leading to faster and more robust bone regeneration. Because PRP is derived from the patient's own blood, it poses no risk of immune reaction while providing a rich osteogenic stimulus at the injury site [42].	PRP therapy creates a growth factor-enriched milieu at nerve injury sites that can enhance regeneration. Platelet α-granules in PRP contain factors like PDGF, VEGF, and EGF, which not only nourish injured neurons and glia but also modulate the immune response to injury [43]. In the early post-injury phase, PRP-released factors attenuate pro-inflammatory signals and promote macrophage polarization toward the M2 macrophage healing phenotype [43]. The PRP fibrin clot itself provides a transient scaffold that supports cell migration and axon sprouting. Additionally, high levels of VEGF from PRP improve revascularization of the regenerating nerve, ensuring adequate blood supply for metabolically demanding regrowing axons [43]. Together, these actions translate into improved axonal regeneration and functional recovery in preclinical nerve injury models.

Applications in bone and nerve repair

Bone Repair

ECM components and perinatal-derived biomaterials, such as the AM, placenta, and umbilical tissues, play a significant role in bone regeneration. The human AM, the innermost layer of the placenta, is rich in collagens, growth factors, and stem cells, making it a biocompatible scaffold for osteogenesis [29]. Due to its bioactive ECM and cellular content, AM can modulate inflammation and fibrosis while promoting new bone formation [29]. Preclinical studies have suggested that applying human amniotic fluid or AM to bone defects accelerates healing. For example, a preclinical in vivo experimental animal study demonstrated that injecting amniotic fluid into rabbit calvarial defects significantly increased ossification and bone density compared to controls [44]. Similarly, a systematic review of in vivo pre-clinical and clinical studies involving AM for bone regeneration suggested that covering bone grafts with AM in animal models leads to faster maturation of bone tissue [29].

Clinically, ECM and perinatal biomaterials are being integrated into common orthopedic and dental procedures for bone repair. In bone grafting and oral surgery, AM allograft membranes have been used for guided bone regeneration (GBR) in periodontal and jawbone defects. Studies in patients with periodontal bone loss showed that adding an AM membrane over grafts leads to improved bone fill and clinical attachment levels at 6-12 months [29]. Notably, AM membranes performed comparably to standard collagen membranes in GBR with no reported adverse effects [29]. In spinal fusion surgery, perinatal tissue allografts have been explored as orthobiologics to enhance fusion and healing [45]. A systematic review of the use of AM in spinal pathologies demonstrated that combining amniotic fluid with bone allograft significantly increased spinal fusion rates versus allograft alone in animal models of lumbar fusion [46]. Early clinical use in spine surgery has also been reported. For example, applying a cryopreserved AM around the surgical site during lumbar discectomy or fusion reduced postoperative fibrosis and improved functional outcomes [46]. In fracture management, perinatal derivatives show promise as adjuncts to stimulate repair. Experimental fracture studies found that defects treated with human amniotic fluid exhibited more rapid and robust bone regeneration than fractures without this adjunctive treatment [44]. Although human trials are still limited, these preclinical and early clinical findings support the incorporation of ECM-based scaffolds and amniotic derivatives into orthopedic reconstructive surgery to improve bone healing outcomes.

Nerve Repair

ECM scaffolds and amniotic-derived biomaterials have similarly been applied to nerve injury repair, capitalizing on their ability to support neural regeneration. Decellularized nerve allografts are a prime example of ECM-based conduits used in clinical peripheral nerve repair. These grafts retain the native nerve ECM architecture (collagens, laminin tubes) and can guide axonal regrowth without provoking immune rejection. In an early clinical study of hand nerve repair, decellularized allografts were used to reconstruct sensory defects, showing promising functional recovery in small segmental gaps without the need for autografts [47]. A recent multicenter randomized controlled trial comparing decellularized allograft to hollow collagen conduits in digital nerve injuries found both achieved similar recovery in short gaps, but the allograft yielded superior outcomes in larger gaps [48]. This highlights how an intact ECM scaffold may provide a more permissive bridge for axons than purely synthetic tubes in challenging nerve repairs.

Perinatal tissues, such as the AM, have been applied as adjuncts in peripheral nerve repair and neurosurgical interventions to enhance neural healing. The AM can be wrapped around sutured nerves as a biological membrane to prevent scarring and provide trophic support. Animal studies and early human trials have suggested that AM wrapping around a repaired nerve limits perineural adhesions and neuroma formation, creating a favorable environment for regeneration [49,50]. The AM's avascular stromal matrix contains anti-inflammatory cytokines and neurotrophic factors that modulate the injury site by reducing fibrosis, inhibiting pro-inflammatory cytokine release, and supporting neural cell differentiation [49,50]. In a rodent model of nerve transection, AM wrapping coincided with faster functional recovery, supported by improved motor function and nerve conduction. This was also associated with upregulation of growth-associated proteins and neurotrophic factors such as NGF and brain-derived neurotrophic factor (BDNF) in the regenerating nerve [30]. Clinically, a pilot trial demonstrated that using an AM allograft to cover a repaired peripheral nerve resulted in improved functional recovery and reduced scar tethering, with investigators attributing these outcomes to the AM's protective and regenerative properties [50].

ECM and amniotic-derived biomaterials are also being investigated in spinal cord injury (SCI) and other neurosurgical repair scenarios. Injectable ECM hydrogel scaffolds derived from tissues such as the spinal cord or bladder have shown potential to fill lesion cavities and support neural tissue regrowth in SCI models [51]. These hydrogels integrate into injured spinal cord tissue, promoting neovascularization and some axonal infiltration, while modulating the local immune response toward a reparative phenotype. Although these strategies remain preclinical, they highlight the potential of biomimetic ECMs to provide both structural and biochemical support for central nervous system repair.

In neurosurgery, human AM grafts have been used as natural dural substitutes and adhesion barriers. For example, in a preclinical rat model, dried AM patches were applied to repair dural defects after cranial surgery, and findings suggested they restored the dural layer and were gradually replaced by native tissue over time [52]. In spinal tethered cord release procedures, placement of an AM or chorion sheet over the surgical site has been associated with reduced postoperative fibrosis and potentially lower rates of re-tethering, as suggested by preliminary clinical studies [46]. These applications leverage the AM's anti-fibrotic and immunomodulatory properties to improve outcomes in neurosurgical interventions. Table 3 provides an overview of current procedural applications of ECM and amniotic derivatives across various clinical and preclinical contexts in bone and nerve repair.

**Table 3:** Procedural Applications of Extracellular Matrix and Amniotic Derivatives in Bone and Nerve Repair AM: amniotic membrane; GBR: guided bone regeneration; ECM: extracellular matrix; FDA: Food and Drug Administration; dHACM: dehydrated amniotic membrane allograft

Application (Bone or Nerve)	Biologic Type (Source)	Status (Clinical or Preclinical)
Periodontal/Alveolar Bone Grafting - Guided bone regeneration in dental implants and periodontics [29]	Amniotic membrane allograft (placental AM) as GBR scaffold	Clinical (trials in patients)
Spinal Fusion - Enhancing arthrodesis in lumbar/cervical fusion surgery [46]	Amniotic fluid concentrate injection; amniotic membrane patch (amnion/chorion)	Clinical (pilot studies) + preclinical evidence
Long Bone Fracture Healing - Treatment of critical-size bone defects or non-unions [44]	Amniotic fluid or placental tissue (injectable ECM, stem cell-rich fluid)	Preclinical (animal models)
Peripheral Nerve Repair (Gap Bridging) - Repair of segmental nerve injuries in limbs [47,48]	Decellularized nerve ECM allograft (processed human nerve)	Clinical (FDA-approved allograft)
Peripheral Nerve Wrap - Adjunct cover for neurorrhaphy or nerve graft [49,50]	Amniotic membrane (amnion/chorion) sheet allograft	Clinical (case series/trial)
Spinal Cord Injury Repair - Scaffold for regenerating transected or damaged spinal cord [51]	Injectable ECM hydrogel (decellularized spinal cord or bladder matrix)	Preclinical (animal studies)
Dural Repair in Neurosurgery - Patch graft for dura mater after cranial or spinal surgery [52]	Dehydrated amniotic membrane allograft (dHACM)	Clinical (case reports)
Prevention of Epidural Adhesions - e.g., after laminectomy, discectomy, or tethered cord release [46]	Dual-layer amniotic membranes	Clinical (small clinical trials, case series, feasibility studies)

Preclinical evidence

Bone Regeneration

Pre-clinical studies of amniotic-derived products for orthopedic treatments have demonstrated promising results, establishing a solid foundation for future human clinical trials [29,26]. Current literature has demonstrated the ECM derivatives' abilities to enhance bone regeneration in animal models. In rat tibial fracture studies, wrapping the fracture location with cryopreserved human AM improved healing, yielding higher histologic scores of new bone and larger callus formation post-injury compared to controls [53]. Similarly, covering a critical-size femoral bone defect in rats with AM led to significantly greater new bone formation than an unprotected defect, supported by upregulation of osteogenic and remodeling genes, including CXCR4, MCP-1, osteocalcin, and Cathepsin K [54].

Recent advances have further validated these findings. Fenelon et al. reported that a BMP-2 scaffold with amnion wrap achieved bone healing equivalent to a two-step Masquelet membrane technique in a rat femoral defect, simplifying the procedure [55]. In a rabbit distraction osteogenesis model, fresh AM accelerated consolidation with bridging by four weeks and corticalization by eight weeks compared to controls [56]. Notably, Priddy et al. found that AM wrapping reduced heterotopic ossification in rat femoral defects by acting as a barrier to excessive bone growth while maintaining defect union by 12 weeks when combined with BMP-2 and collagen grafts [57].

Specialized applications have also shown promise. In rat supraspinatus repair, applying decellularized AM at the tendon-bone interface increased bone formation, ultimate failure load, and histologic healing at four weeks [58]. Additionally, a bovine AM-hydroxyapatite composite implanted in rat tooth extraction sockets increased collagen deposition, osteoblast proliferation, and expression of BMP2, RUNX2, and osteocalcin at 14 and 28 days [59].

Peripheral Nerve Repair

Both in vitro and in vivo studies have demonstrated the recovery effects of perinatal ECM grafts on peripheral nerve injury. The AM has long been recognized for its anti-inflammatory properties, offering significant advantages in nerve repair by inhibiting scar formation [60]. In an 8-mm rat sciatic nerve gap, wrapping a collagen-filled nerve conduit with fresh human AM improved axonal regeneration, demonstrating significantly shorter nerve latency, larger compound muscle action potentials, thicker myelinated axons, and improved g-ratio compared to conduit alone [61].

Wolfe et al. reported that both human amnion wraps and standard collagen nerve wraps enhanced nerve healing compared to no wrap in a rodent sciatic nerve injury model, but the improvements were greatest with the AM wrap, yielding higher axon counts, thicker myelin, and better muscle reinnervation [26]. Advanced bioengineered approaches have further enhanced these outcomes. A polycaprolactone (PCL)/AM nanofiber wrap promoted in vitro SH-SY5Y axonal growth and, in vivo, increased anti-inflammatory M2 macrophages, elevated IL-10/IL-13, reduced IL-6/TNF-α, and enhanced myelination and axonal regeneration [62].

Recent studies have explored additional therapeutic combinations. In a rat sciatic crush injury model, a decellularized human AM hydrogel improved functional indices (Sciatic Functional Index), latency, and remyelination markers (NF-200, S-100β), with additional benefit when combined with metformin [63]. A gelatin nanofiber-reinforced decellularized amnion composite reduced fibrotic scarring and improved axonal and functional recovery in rat peripheral nerve injury [60].

The preclinical evidence regarding the use of AM-derived products in orthopedic applications, particularly in bone regeneration and peripheral nerve repair, holds significant promise for advancing regenerative medicine. The results of these animal model studies consistently demonstrate the potential of AMs to enhance tissue healing, reduce fibrosis, and promote favorable cellular environments for bone and nerve regeneration. These findings, while promising, highlight the need for further research, particularly in human clinical trials, to confirm the safety, efficacy, and long-term benefits of these biologic products in patients undergoing orthopedic procedures. The clinical implications of these findings suggest a paradigm shift in how orthopedic surgeons might approach tissue repair, moving toward biologically driven solutions that harness the body's regenerative capacity. As we transition to examining clinical outcomes, it is crucial to explore how these preclinical results manifest in human patients. Table 4 provides a comprehensive overview of key preclinical studies examining AM and ECM derivatives in bone and nerve repair, detailing study models, interventions, outcome measures, and primary findings across both therapeutic applications.

**Table 4:** Summary of Preclinical Studies of Amniotic Membrane and ECM Derivatives in Bone and Nerve Repair HAM: human amniotic membrane; CXCR-4: C-X-C motif chemokine receptor 4; MCP-1: monocyte chemoattractant protein-1; OC: osteocalcin; CatK: cathepsin K; CPC/BMP2: calcium phosphate cement/bone morphogenetic protein-2; rhBMP-2: recombinant human bone morphogenetic protein-2; dHACM: dehydrated human amnion/chorion membrane; NCT: normal control; OSR: only suturing repair; DAM: decellularized amniotic membrane; BAM-HA: bone-attached matrix with hyaluronic acid; RUNX2: runt-related transcription factor 2; Gel-dAM: gelatin nanofiber-decellularized amniotic membrane composite; PGA-collagen tube: polyglycolic acid-collagen tube; PGA-c/HAM: polyglycolic acid-collagen/human amniotic membrane; dAM: decellularized amniotic membrane; SFI: sciatic functional index; SSI: Sensory Scoring Index; latency time: nerve conduction latency time; remyelination rate: rate of axonal myelin regeneration; NF-200: neurofilament 200; S-100β: S-100 beta protein; PCL/AM: polycaprolactone/amniotic membrane; SH-SY5Y cells: human neuroblastoma cells; M2 macrophage: anti-inflammatory macrophage subtype; IL-10: interleukin-10; IL-13: interleukin-13; IL-6: interleukin-6; TNF-α: tumor necrosis factor alpha

Study Reference	Application Area	Model Type	Intervention	Product Used	Findings
Sarı et al., 2019 [53]	Fracture Healing	Wistar-Albino rats (n=28)	HAM wrapping around tibial fracture site + K-wire fixation vs. K-wire fixation alone	Cryopreserved human amniotic membrane (HAM)	HAM significantly improved fracture healing with better histological scores, larger callus formation, and accelerated bone formation. The HAM group achieved complete woven bone formation by six weeks vs. partial healing in the control group.
Tang et al., 2018 [54]	Bone Regeneration	Bone marrow mesenchymal stem cells (in vitro), rat femoral defect model (in vivo)	Human acellular amniotic membrane implantation in femoral defects	De-epithelialized and lyophilized human acellular amniotic membrane	Human acellular amniotic membrane demonstrated osteoinductive properties, enhanced bone marrow MSC proliferation and osteogenic differentiation, and significantly improved bone regeneration with increased expression of bone remodeling genes (CXCR-4, MCP-1, OC, CatK).
Fenelon et al., 2021 [55]	Bone Regeneration	Human BMSCs (in vitro), Sprague Dawley rats femoral defect model (in vivo)	5 groups: Empty, CPC/BMP2 scaffold alone, CPC/BMP2 + lyophilized HAM, CPC/BMP2 + decellularized/lyophilized HAM, CPC/BMP2 + induced membrane (Masquelet)	3D-printed calcium phosphate cement scaffold loaded with 10μg rhBMP-2; lyophilized and decellularized/lyophilized HAM	CPC/BMP2 scaffold alone achieved satisfactory bone healing without requiring membrane coverage. No significant difference between the Masquelet technique and the single-step HAM approach. Decellularized/lyophilized HAM performed better than lyophilized HAM alone.
Elmounedi et al., 2025 [56]	Distraction Osteogenesis	New Zealand white rabbits (n=10)	Tibial osteotomy with rapid distraction lengthening (2.8 mm/day) with fresh HAM implantation vs. distraction alone	Fresh human amniotic membrane (HAM)	The HAM group showed complete bone consolidation with homogeneous callus formation at 4 weeks and complete corticalization at 8 weeks. Histological analysis revealed thick, continuous cortices with healthy intramedullary tissue. HAM enhanced bone consolidation during rapid distraction osteogenesis.
Priddy et al., 2023 [57]	Heterotopic Ossification Prevention	Sprague-Dawley rats (n=11) femoral segmental defect model	High-dose BMP-2 (30 μg) in collagen sponge alone vs. collagen sponge surrounded by amniotic membrane	Dehydrated human amnion/chorion membrane (dHACM) allograft (AmnioFix®)	Amniotic membrane significantly reduced heterotopic ossification compared to collagen sponge alone while maintaining equivalent bone formation within the defect by 12 weeks. AM retained more BMP-2 than synthetic membranes and provided spatial control of bone regeneration.
Wang et al., 2025 [58]	Rotator Cuff Repair/Tendon-Bone Integration	Sprague-Dawley rats (n=33)	3 groups: Normal control (NCT), suturing repair only (OSR), suturing repair with DAM placed between the supraspinatus tendon and bone (DAM)	Decellularized amniotic membrane (DAM) prepared from fresh amniotic membrane	The DAM group showed significantly higher ultimate load to failure, increased new bone volume, and superior histological evaluation compared to the OSR group. Despite a dense basement layer, DAM enhanced tendon-bone healing rather than impeding it, though still inferior to a normal intact rotator cuff.
Octarina et al., 2024 [59]	Ridge Preservation	Sprague-Dawley rats (n=30)	5 groups: Control (no treatment), xenograft, BAM-HA (2:3), BAM-HA (3:7), BAM-HA (7:13) applied in extraction sockets	Bovine amniotic membrane-hydroxyapatite (BAM-HA) composites in three different ratios	BAM-HA significantly increased collagen density, osteoblast proliferation, and expression of BMP2, RUNX2, and osteocalcin compared to control. Higher hydroxyapatite content enhanced bone formation markers. Results suggest BAM-HA can be used for ridge preservation to prevent bone resorption following tooth extraction.
Chen et al., 2023 [60]	Peripheral Nerve Injury Repair	Animal model (specific details not provided in abstract)	Gelatin nanofiber-decellularized amniotic membrane composite (Gel-dAM) vs. controls for peripheral nerve injury repair	Decellularized amniotic membrane reinforced with gelatin nanofiber membrane through interfacial bonding	Gel-dAM composite showed enhanced mechanical properties, reduced degradation rate, while retaining the bioactivity of dAM. Led to improved axon regeneration, reduced fibrotic response, and better functional recovery compared to controls. Represents a novel tissue engineering approach for peripheral nerve repair.
Iwao et al., 2023 [61]	Peripheral Nerve Injury Repair	Rats with sciatic nerve 8-mm defect model (n=15)	3 groups: PGA-collagen tube alone, PGA-collagen tube + fresh HAM wrapping, Sham control	Fresh human amniotic membrane (HAM) wrapped around a polyglycolic acid tube filled with collagen (PGA-c)	PGA-c/HAM group showed significantly better recovery vs. PGA-c alone: improved terminal latency (3.4±0.31 vs. 6.6±0.72 ms), compound muscle action potential (0.19±0.025 vs. 0.072±0.027 mV), myelinated axon perimeter (15±1.3 vs. 8.7±0.63 μm), and g-ratio (0.69±0.0089 vs. 0.78±0.014). Combined application highly promotes peripheral nerve regeneration.
Dolatabadi et al., 2025 [63]	Peripheral Nerve Injury Repair	Male Wistar rats (n=60, 12 per group), sciatic nerve compression model	5 groups: Control, model (compression only), hydrogel (dAM hydrogel injection into epineurium), metformin (200 mg/kg IP), Mix (hydrogel + metformin)	Decellularized amniotic membrane (dAM) hydrogel + metformin	Hydrogel derived from decellularized amniotic membrane improved functional nerve recovery when injected into the epineurium. SFI, SSI, latency time, remyelination rate, and expression of NF-200 and S-100β improved in the hydrogel group. Metformin administration reinforced therapeutic effects when combined with hydrogel, enhancing response to mechanical stimuli, myelin density, and axonal regeneration.
Liu et al., 2023 [62]	Peripheral Nerve Injury Repair	SH-SY5Y cells (in vitro), animal nerve injury model (in vivo)	Electrospun nanofibrous polycaprolactone/amniotic membrane (PCL/AM) composite vs. controls	Multi-layer nanofibrous polycaprolactone/amniotic membrane composite fabricated using electrospinning	PCL/AM promoted axon growth and neuronal differentiation in vitro, enhanced M2 macrophage polarization, increased anti-inflammatory factors (IL-10, IL-13), decreased pro-inflammatory factors (IL-6, TNF-α), and promoted myelin sheath and axon regeneration. Composite regulated inflammatory microenvironment to enhance nerve repair.

Clinical evidence

Orthopedic Applications

Much of the evidence surrounding amniotic and perinatal tissue matrices demonstrates that these products can be used as barrier membranes or graft additives to enhance bone repair, especially in cases where enhanced osteogenesis or reduced inflammation is desired [64]. Decellularized bone allograft is a purified ECM scaffold that has previously been used to repair significant bone defects with reliable success. Newer ECM-based adjuncts are being translated into clinical practice with promising results.

For spinal applications, a retrospective clinical study of lumbar spinal fusion found that adding an amniotic suspension allograft (ASA) (NuCel®) to bone graft achieved improved spinal fusion rates of approximately 99% at 12 months [29]. Recent clinical evidence has also emerged for the application of hand surgery. A clinical case series reported that eight patients with atrophic metacarpal or phalangeal nonunions treated with iliac micro-grafts plus human AM wrapping achieved radiographic union by approximately two months and excellent functional outcomes [65].

Injectable amniotic allografts have shown significant benefits in knee osteoarthritis (OA). In a multicenter level 1 randomized controlled trial, 200 patients with knee OA were randomized to receive a single injection of human amniotic suspension allograft (HASA) versus hyaluronic acid (HA) versus saline [66]. The ASA group demonstrated superior outcomes, with 69.1% of ASA-treated patients achieving OARSI-OMERACT responder rates compared to 39.1% for the HA group and 42.6% for the saline group. Moreover, patient-reported outcomes, including Knee Injury and Osteoarthritis Outcome Score (KOOS) and Visual Analog Scale (VAS) pain scores, showed significantly greater improvement in the ASA group at three and six months compared to either the HA or saline group [66].

Additional studies focusing on amniotic derivatives have confirmed these improvements in knee OA. A prospective pilot study reported on the use of HASA and demonstrated the anti-inflammatory and cartilage regenerative potential of this substance in treating OA [67]. International Knee Documentation Committee (IKDC) and VAS pain scores were significantly improved in patients at three, six, and 12-month follow-up visits for those who received a single intra-articular injection of HASA, with no serious adverse effects reported [67].

Peripheral Nerve Injuries

Clinical applications of amniotic tissue products in peripheral nerve surgery have shown promising early results [68]. In carpal tunnel syndrome, surgeons have begun employing commercially available amnion/chorion nerve wraps with encouraging outcomes. A randomized trial involving 47 wrists found that carpal tunnel release plus AM transplantation significantly improved symptom severity and functional scores at one-year follow-up compared to carpal tunnel release without AM supplementation [69].

For recurrent cubital tunnel syndrome, when treated with neurolysis simultaneously with human AM allograft supplementation, eight patients showed improved postoperative pain levels, grip strength increases of +25 pounds, and reduced VAS/QuickDASH scores by approximately 4.7 points and QuickDASH scores by approximately 40 points at 28.9-month follow-up [70]. Human processed umbilical cord membrane has also been utilized in traumatic nerve reconstruction. A study of 20 patients with ulnar, radial, peroneal, posterior tibial, medial nerve, and nerve of the third digit injuries treated with umbilical cord membrane (Avive®) reported significant VAS and QuickDASH improvements with no repair failures [71].

These various applications of amniotic derivatives in orthopedic surgery demonstrate the significant potential of these products to assist in the restorative process of both bone regeneration and peripheral nerve injuries. The evidence spans from robust preclinical models showing enhanced healing mechanisms to clinical trials demonstrating improved patient outcomes across multiple orthopedic applications. However, further research and application of these derivatives in peripheral nerve injuries and bone regeneration are needed to determine their full benefit in treating a variety of orthopedic and neurological conditions before they are universally applied in clinical practice. Table 5 summarizes the current clinical evidence for AM and perinatal tissue derivatives in orthopedic and neurological applications, highlighting patient populations, treatment protocols, outcome measures, and clinical efficacy across diverse therapeutic indications.

**Table 5:** Clinical Applications and Outcomes of Amniotic Membrane Derivatives in Orthopedic and Neurological Practice AM: amniotic membrane; ASA: amniotic suspension allograft; HASA: human amniotic suspension allograft; dHACM: dehydrated human amnion/chorion membrane; PUCM: processed human umbilical cord membrane; ORIF: open reduction and internal fixation; TAM: total active motion; VAS: Visual Analog Scale; IKDC: International Knee Documentation Committee; OMERACT-OARSI: Outcome Measures in Rheumatology - Osteoarthritis Research Society International; CTRS: carpal tunnel release surgery; AMT: amniotic membrane transplantation; SRN: superficial radial nerve; s2PD: static two-point discrimination; m2PD: moving two-point discrimination; QuickDASH: shortened Disabilities of the Arm, Shoulder, and Hand score; SF-36: Short Form Health Survey - 36 items

Study Reference	Study Type	Application Area	Model Type	Intervention	Product Used	Conclusion
De Francesco et al., 2025 [65]	Case Series (Human)	Bone Regeneration/Nonunion Treatment	8 patients (6 males, 2 females; age range: 22-56 years) with atrophic nonunion of metacarpals and phalanges	Regenerative surgical protocol: autologous bone micro-grafts + fresh human amniotic membrane creating "biological regenerative chamber" + ORIF fixation	Autologous bone micro-grafts (Rigenera® processed, 80 μm diameter) + fresh human amniotic membrane	All patients achieved radiographic consolidation within 1-2 months. Functional outcomes showed excellent/good results with TAM, grip/pinch strength within normative ranges, and minimal pain (VAS ≤2). No complications observed. The regenerative approach was associated with rapid consolidation and excellent functional recovery compared to traditional methods.
Farr et al., 2019 [66]	Randomized Controlled Trial (RCT)	Knee Osteoarthritis Treatment	200 subjects with moderate knee OA (Kellgren-Lawrence grades 2-3), randomized 1:1:1 across 12 sites	Single intra-articular injection comparison: ASA vs. hyaluronic acid (HA) vs. saline control, single-blind design	Amniotic suspension allograft (ASA) - cryopreserved amniotic fluid cells and membrane particulate	ASA demonstrated superior results to both HA and saline controls. Treatment failure rates at 3 months: ASA (13.2%), HA (68.8%), saline (75%). OMERACT-OARSI responder rates at 6 months: ASA (69.1%), HA (39.1%), saline (42.6%). ASA showed significantly greater improvements in pain, function, and quality of life measures compared to controls.
Natali et al., 2022 [67]	Prospective Case Series (Human)	Knee Osteoarthritis Treatment	25 patients with knee OA (Kellgren-Lawrence grades 1-3), mean age 45.09 years, single-arm prospective design	Single intra-articular injection of 3 mL human amniotic suspension allograft (HASA), evaluated at 3, 6, and 12 months	Human amniotic suspension allograft (HASA) - amniotic membrane homogenized into suspension, stored at -80°C	Statistically significant improvement in IKDC and VAS scores at all timepoints vs. baseline (p<0.001). Peak improvement at 6 months with mild regression by 12 months, but still significantly better than baseline. Minor side effects in 16% (transitory burning/mild pain). No severe complications. Treatment appears safe and effective for moderate knee OA.
Buentello-Volante et al., 2020 [69]	Randomized, Single-Blind Controlled Trial	Carpal Tunnel Syndrome (CTS) Treatment	35 patients (47 wrists) with unilateral or bilateral CTS	Carpal tunnel release surgery (CTRS) combined with amniotic membrane transplantation (AMT) vs. CTRS alone	Amniotic membrane transplantation (AMT)	CTRS combined with AMT demonstrated significantly better outcomes than CTRS alone at 1-year follow-up, with reductions in Boston Carpal Tunnel Syndrome Questionnaire, Disabilities of the Arm, Shoulder, and Hand, and Historical-Objective scale scores, indicating improved functional recovery and less recurrence of CTS.
Gaspar et al., 2017 [70]	Retrospective Review with Prospective Follow-Up	Radial Nerve Entrapment (Wartenberg Syndrome) Treatment	3 females with SRN neuropathy	Neurolysis and nerve wrapping using amnion-based allograft adhesion barrier	Amnion-based allograft adhesion barrier	Neurolysis and amnion nerve wrapping resulted in significant improvements in pain (VAS change -4.7) and function (QuickDASH change -40), with no adverse events or reactions. This technique may be effective for perineural scar prevention in SRN neuropathy and warrants further study.
Cox et al., 2021 [71]	Prospective, Single-Center Pilot Study	Traumatic Peripheral Nerve Injury Treatment	20 patients with traumatic peripheral nerve injuries (upper and lower extremities)	Multi-level surgical reconstruction with processed human umbilical cord membrane (PUCM) covering exposed non-transected nerves	Avive™ Soft Tissue Membrane (processed human umbilical cord membrane)	PUCM usage in traumatic peripheral nerve surgery resulted in significant improvements in pain (VAS), sensory recovery (s2PD, m2PD), motor function (grip and pinch strength), and patient-reported outcomes (QuickDASH, SF-36), compared favorably with control groups and historical data, suggesting its potential as a useful adjunct in nerve repair.

Limitations

Regulatory and Safety Concerns

The complexity of ECM and amniotic-derived products creates significant regulatory hurdles. Standardized processing protocols and quality sourcing are essential to ensure consistent efficacy, yet current manufacturing variability limits real-world implementation [72]. The FDA and other regulatory agencies continue adapting frameworks to accommodate these novel biomaterials, but approval pathways remain challenging and time-consuming. Despite decellularization processes, ECM scaffolds may still elicit immune responses due to residual antigens and damage-associated molecular patterns (DAMPs) [73]. While perinatal tissues demonstrate inherent immunomodulatory properties, concerns about long-term immunogenicity and potential acute rejection with repeated exposure require ongoing investigation. Current mitigation strategies, including antigen masking and optimized sterilization, need further validation.

Clinical Evidence Gaps

Limited long-term follow-up data and small sample sizes characterize much of the current clinical evidence [65-67]. Most studies lack adequate statistical power to definitively establish superiority over conventional treatments. The absence of standardized outcome measures across studies makes meaningful comparison difficult, while the heterogeneity of patient populations and treatment protocols limits the generalizability of results.

Access Barriers and Ethical Considerations

Cost represents a major implementation barrier, with ECM and amniotic derivatives commanding substantial price premiums over conventional alternatives. Limited insurance coverage and reimbursement uncertainties compound these challenges, while the absence of clear cost-effectiveness data across diverse clinical indications creates surgeon hesitancy. Cost-effectiveness analyses have already shown economic favorability of AM therapies in ophthalmology as well as diabetic foot ulcer management [74,75]. These factors risk creating healthcare disparities where advanced regenerative therapies remain accessible only to well-resourced patients and institutions.

Perinatal tissue sourcing raises important ethical questions regarding informed consent, commercialization, and equitable access. Robust protocols must ensure expectant mothers understand tissue donation implications without coercion, while the substantial commercial value of processed products raises concerns about fair distribution and preventing two-tiered healthcare systems. Long-term patient follow-up and data security represent ongoing ethical obligations requiring careful attention.

Future directions

Technological Advancement

Integration of emerging technologies offers significant potential for enhancing ECM and amniotic derivatives. Efforts to combine various cell types of ECM have shown success in bone regeneration by promoting cell-to-cell communication and regulation of regenerative behavior [76]. Amniotic cells appear to modulate and suppress host immune cells, but with several passes, immunosuppression was lost and acute rejection occurred [77]. Three-dimensional bioprinting combined with artificial intelligence-guided scaffold design could enable patient-specific constructs with optimized mechanical properties and bioactive factor release kinetics [78]. Advanced manufacturing techniques may address current limitations in cell-specific properties and degradation rates while enabling scalable production.

Clinical Research Priorities and Translational Implementation

Future research should prioritize deeper mechanistic insights into ECM-cell interactions, particularly how scaffold topography, stiffness, and biochemical composition influence lineage commitment and tissue integration. Understanding the longevity of immunosuppressive properties and developing strategies to maintain therapeutic efficacy across multiple passages will be crucial for clinical translation. Biomarker discovery to predict treatment success based on patient-specific factors (age, comorbidities, immune status) could enable personalized therapeutic approaches.

Well-designed comparative effectiveness studies through multi-center randomized controlled trials are urgently needed. These studies require adequate sample sizes (n=200-400 per arm), combined objective and patient-reported outcomes, and long-term registry monitoring of 5-10 year safety and effectiveness data.

Successful clinical adoption requires addressing fundamental barriers through interdisciplinary collaboration between orthopedic surgery, regenerative biology, and bioengineering. Standardization of tissue sourcing, processing protocols, and outcome reporting must be prioritized alongside the development of evidence-based clinical guidelines. Cost-effectiveness analyses and real-world outcome studies are essential for establishing reimbursement frameworks and demonstrating clinical value. Only through systematic addressing of these challenges can the promising potential of ECM and perinatal-derived biomaterials be translated into consistent, accessible clinical benefits for patients with complex bone and nerve injuries.

## Conclusions

Several critical gaps must be addressed to optimize ECM-based scaffolds for clinical use. Future preclinical research should focus on defining optimal scaffold composition, degradation rates, and mechanical properties for specific applications, while investigating molecular-level biomaterial-cell interactions to understand how ECM topography and stiffness influence lineage commitment. Clinical translation requires comparative effectiveness trials against current standards, such as randomized controlled trials comparing AM-augmented nerve repair versus autologous grafts in larger defects. Long-term success also depends on comprehensive cost-effectiveness analyses and registry studies to evaluate economic viability, real-world outcomes, and safety profiles over time, helping overcome reimbursement barriers.

While ECM and perinatal-derived biomaterials show remarkable regenerative potential, their clinical utility ultimately depends on rigorous, mechanistically informed translational research that addresses material optimization, patient selection, long-term outcomes, and economic considerations to advance toward reliable, biologically driven solutions for complex musculoskeletal and neurological injuries.
